# Skin Findings in a Patient with Sjogren's Syndrome

**DOI:** 10.1155/2016/4829459

**Published:** 2016-09-08

**Authors:** Prajwal Boddu, Abdul S. Mohammed, Sonali Khandelwal

**Affiliations:** ^1^Advocate Illinois Masonic Medical Center, Department of Internal Medicine, 856 West Nelson Street, Apt No. 2002, Chicago, IL 60657, USA; ^2^Advocate Illinois Masonic Medical Center, Department of Internal Medicine, 2356 North Elston Avenue, No. 306, Chicago, IL 60614, USA; ^3^RUSH University Medical Center, Department of Rheumatology, 1725 West Harrison Street, Chicago, IL 60612, USA

## Abstract

Hypergammaglobulinemic purpura (HGP) is a syndrome constellating recurrent purpura, hypergammaglobulinemia, positive rheumatoid factor (RF), anti-Ro/La antibodies, and elevated erythrocyte sedimentation rate (ESR). We present a case of a 29-year-old female who was diagnosed with Sjogren's syndrome four years prior to presenting with rash on her lower extremities for a period of 6 months. Skin biopsy at the initial visit was consistent with leukocytoclastic vasculitis and was initiated on treatment for it. Her rash evolved into 2–5 mm scattered purpurae while she was on the treatment and a repeat biopsy showed extravasation of RBCs, a sparse mononuclear infiltrate with deposition of plasma cells, and no evidence of leukocytoclastic vasculitis, thus showing a transition from neutrophilic to mononuclear inflammatory vascular disease which is a rare occurrence. Hypergammaglobulinemic purpura sometimes turns out to be a challenging disease to manage and requires an integrated effort from the primary care doctors, rheumatologist, and dermatologist.

## 1. Introduction

Hypergammaglobulinemic purpura (HP) is a syndrome constellating recurrent purpura, hypergammaglobulinemia, positive rheumatoid factor (RF), anti-Ro/La antibodies, and elevated erythrocyte sedimentation rate (ESR) [1]. The purpura usually manifests on the lower part of the body after prolonged standing. HP could be primary or be associated with other connective tissue diseases, including Sjogren's syndrome [2, 3]. Two different types of inflammatory vascular diseases have been reported in Sjogren's which are neutrophilic inflammatory vascular disease (NIVD) and mononuclear inflammatory vascular disease (MIVD). Here we report a case demonstrating transition of vasculitis from NIVD to MIVD in serial biopsies, a rare and unusual occurrence.

## 2. Case Report

**Figure 1:**
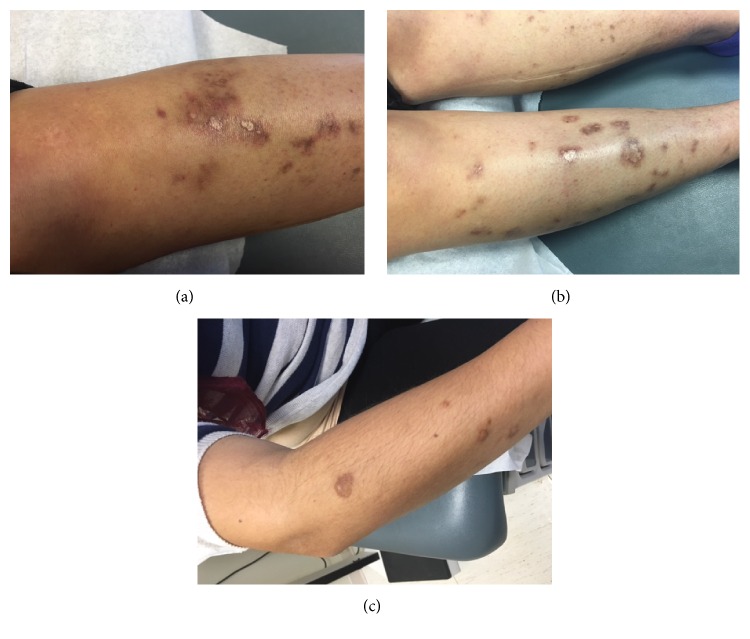
Pictures demonstrating rash in our patient.

A 29-year-old female with history of Sjogren's syndrome for the past 4 years presented to our hospital with complaints of rash over the lower extremities over a period of 6 months. Her initial presentation included symptoms of diffuse joint pain, dry eyes, dry mouth, and parotid gland swelling. Her laboratory findings at that time were significant for mild anemia (Hg 12.5 g/dL), mild leukopenia (3.9/mm^3^), elevated SSA and SSB titers, and ESR of 40 mm/hr. The patient first observed a petechial rash six months ago, which had progressively gotten worse to involve both lower extremities and the right upper extremity (refer to Figures 1(a), 1(b), and 1(c)). A skin biopsy performed at the initial onset of symptoms was suggestive of findings consistent with leukocytoclastic vasculitis for which she was treated with steroids and hydroxychloroquine. However, her rash spread while being on both these agents to evolve into 2–5 mm scattered purpurae involving both the legs and right forearm. Labs during this visit were remarkable for a total protein of 9 g/dL, globulin of 5.5 g/dL, albumin of 3.5 g/dL, ESR of 75 mm/hr, C 3 of 84 gm/dL, C 4 of 7 mg/dL, rheumatoid factor of 95 IU/mL, IgG of 3710 mg/dL, IgA of 553 mg/dL, Kappa of 3.5 mg/dL, and lambda of 2.16 mg/dL. The following labs were either normal or within normal range: complete blood counts, comprehensive metabolic panel, hepatitis panel, and cryoglobulins. A repeat skin biopsy was performed which showed extravasation of RBCs, a sparse mononuclear infiltrate with deposition of plasma cells, and no evidence of leukocytoclastic vasculitis. She was diagnosed with Waldenstrom's hypergammaglobulinemic purpura based on constellation of lower extremity purpura, an elevated erythrocyte sedimentation rate (ESR), and gammopathy.

## 3. Discussion

Hypergammaglobulinemic purpura of Waldenstrom (HGP) was first described by physician Waldenstrom in a series of three patients presenting with recurrent purpura, elevated erythrocyte sedimentation rate, and increased gamma globulinemic fractions. HGP can be primary when it occurs alone or secondary in association with other autoimmune diseases like Sjogren's, SLE, and RA [4]. Sjogren's syndrome is systemic autoimmune disorder characterized by involvement and damage of salivary and lacrimal glands causing sicca symptoms [5]. However, Sjogren's syndrome can present with various extraglandular manifestations including the skin. Cutaneous manifestations, due to small and medium vessel vasculitis, can manifest as varying presentations from petechiae to extensive ecchymotic purpurae. Purpurae appear as crops of pink or red lesions which later turn brown with hemosiderin deposition, as was seen in our patient [6]. An elegant retrospective study by Parodi et al., which looked at 62 patients, reported cutaneous vasculitis in 30.6% of primary SS and in 29.3% of secondary SS cases [7]. A multicenter study distinguished two types of Sjogren's purpura, based on distinct pathophysiological phenomena, into cryoglobulinemic vasculitic purpura, a prelymphomatous condition associated with complement consumption, and HGP, a benign condition, associated with increased globulin fraction from polyclonal B cell activation [8].

Histologically, two types of inflammatory vasculitis have been described in Sjogren's syndrome: the neutrophilic inflammatory vascular disease (NIVD) which is characterized by neutrophil fragmentation and extravasation and fibrinoid necrosis and mononuclear inflammatory vascular disease (MIVD) characterized by mononuclear infiltration of blood vessels [9]. NIVD, though indistinguishable from MIVD in clinical expression, is associated with positive antinuclear antibodies, rheumatoid factor, antibodies to Ro (SS-A), and hypocomplementemia [9]. Serial skin biopsies performed to assess temporal evolution suggest that most patients retain the same histopathological type or show transition from MIVD to NIVD, but not the converse. These findings are supported by similar observations in murine models [9, 10]. These observations led to the proposal of reversed temporal evolution of inflammatory vasculitis in Sjogren's syndrome, contrary to the traditional models of inflammatory vascular disease. It was interesting to note that serial biopsies in our patient documented a temporal transition from NIVD to MIVD, an unexpected pathological finding. However, it must be noted that some patients may have evidence of both histologies on separate sites at the same time [10]. This may partly explain the finding of different histologies at varying times and examined sites of vessel involvement as was observed in our patient.

Treatment for HGPW secondary to autoimmune diseases is not standardized. A case report published by Habib and Nashashibi in 2004 reported two cases of HGP in two sisters with Sjogren's syndrome who responded to colchicine [11]. Colchicine inhibits the polymerization of microtubules by binding their protein subunits and preventing their aggregation [12]. It is not clear how this prevents the development of purpura in HP. Another study published in 1995 looking at patients with HGP secondary to rheumatological diseases reported that indomethacin and hydroxychloroquine are of value in treatment of milder hypergammaglobulinemic purpura and that prednisone should be reserved for severe forms of HGP [13]. A recent case report published in 2013 reported the use of rituximab for recurrent HGP for patient who was on treatment with hydroxychloroquine and azathioprine [14]. HGP sometimes turns out to be a challenging disease to manage and requires an integrated effort from the primary care doctors, rheumatologist, and dermatologist.
